# Effectiveness of Tong-Xie-Yao-Fang combined with Si-Ni-San for irritable bowel syndrome: A protocol for systematic review and meta-analysis

**DOI:** 10.1097/MD.0000000000025198

**Published:** 2021-03-19

**Authors:** Jiawang Jiang, Yun Chen, Ziyi Hu, Huaiyu Li, Jing Ye, Zhiying Yu, Haiyi Tang

**Affiliations:** a Jiangxi University of Traditional Chinese Medicine, Nanchang; b First Affiliated Hospital of Gannan Medical University, Ganzhou; c The Affiliated Hospital of Jiangxi University of Traditional Chinese Medicine, Nanchang, China.

**Keywords:** irritable bowel syndrome, protocol, Si-Ni-San, systematic review, Tong-Xie-Yao-Fang

## Abstract

**Background:**

Irritable bowel syndrome (IBS) has a high morbidity rate worldwide, but there are no effective treatment measures, which seriously affect people's lives. Previous clinical studies on Tong-Xie-Yao-Fang (TXYF) combined with Si-Ni-San (SNS) in the treatment of IBS have been increasing, but there is no systematic evaluation. This study aims to systematically study the effectiveness of TXYF combined with SNS in the treatment of IBS.

**Methods:**

The PubMed, EMBASE, Science Network, Cochrane Library, Chinese Biomedical Literature, Wanfang Chinese Digital Journal and Conference Database, China National Knowledge Infrastructure Database and VIP China Science and Technology Journal Database (VIP) will be used Search related literature, and the search time is from the date of establishment to February 2021. The National Institutes of Health clinical registry Clinical Trials, International Clinical Trials Registry Platform and the Chinese clinical trial registration platform will be searched to find ongoing or unpublished trials. After screening the literature according to the criteria, two researchers independently extracted data according to a predetermined table. The primary outcome is total effective rate. The RevMan 5.3.5 software will be used for statistical analysis. Finally, the recommendation, evaluation, development and evaluation system will be used to evaluate the quality evidence for each result.

**Results:**

This study will provide the latest evidence of efficacy for the TXYF combined with SNS for IBS.

**Conclusion:**

The effectiveness of TXYF combined with SNS for IBS will be evaluated.

**Unique INPLASY number:**

INPLASY202120075.

## 1 Introduction

Irritable bowel syndrome (IBS) is a functional digestive system disease characterized by bowel habits, bloating, abdominal pain and changes in stool characteristics, without organic disease, and belongs to one of gastrointestinal neurosis.^[1,2]^ The pathogenesis of IBS may be closely related to intestinal insensitivity, visceral hypersensitivity, intestinal mucosal activation, and increased intestinal permeability.^[3,4]^ The Rome IV standard is currently an IBS diagnostic standard agreed upon by experts in functional gastrointestinal diseases. According to epidemiological studies, the prevalence of IBS worldwide is about 5%-10%.^[5]^ The annual medical costs related to IBS in Europe are estimated to be about 8 billion euros, China is about 123 billion yuan, and the United States is about 10 billion or more.^[6]^

The main purpose of current treatment of IBS is to improve abdominal pain and bowel habits. Common treatment measures include changing eating habits, taking soluble fiber and antispasmodic drugs.^[7]^ In patients with severe symptoms, low-dose tricyclic antidepressants, intestinal secretions, and antibiotics are often used.^[8,9]^ Due to the lack of indicators such as bacteriological and biochemical abnormalities in IBS, there is no ideal cure drug and symptomatic treatment not only has limited efficacy, but also has a high recurrence rate.^[10]^ Based on the current situation, there is an urgent need to find an effective treatment for IBS.

At present, complementary and alternative therapies are getting more and more attention from IBS patients and clinicians, including traditional Chinese medicine therapies represented by Chinese herbal medicine.^[11,12]^ Tong-Xie-Yao-Fang (TXYF) and Si-Ni-San (SNS) are two ancient formulas that are widely used in the treatment of IBS in China.^[13,14]^ TXYF can improve visceral hypersensitivity in rats, and its mechanism may be related to the up-regulation of TAGLN and Aldh2 and the down-regulation of CK8.^[15]^ Moreover, it can improve intestinal permeability and intestinal mucosal barrier function, which may be related to the inhibition of inflammatory cascade and NF-κB and Notch signaling pathway.^[16]^ The multifunctional synergistic effect of SNS on IBS, including inflammatory response regulation, oxidative stress suppression regulation, and hormone and immune regulation, which may be related to Prostaglandin G synthase-2, Calmodulin-2 and other protein.^[17]^

In summary, the effectiveness of TXYF combined with SNS in the treatment of IBS is worth studying. Although there have been some randomized controlled trials (RCTs) of TXYF combined with SNS in the treatment of IBS, there is no evidence-based medicine to support it. Therefore, we will collect all relevant RCT data for meta-analysis to evaluate the efficacy of TXYF combined with SNS in the treatment of IBS and provide the best available evidence for it.

## 2 Objectives

To evaluate the efficacy of TXYF combination with SNS, in people with IBS. And provide the latest evidences of evidence-based medicine for the clinical treatment of IBS.

## 3 Methods

### 3.1 Study protocol and registration

The protocol has been registered by us on the INPLASY website (registration number: INPLASY202120075: https://inplasy.com/inplasy-2021-2-0075/). The protocol of our study will strictly follow the Preferred Reporting Items for Systematic Review and Meta-Analysis Protocols Statement Guidelines for systematic reviews and meta-analysis.^[18]^

### 3.2 Study search

The PubMed, EMBASE, Web of Science, the Cochrane Library, China Biomedical Literature, the Wanfang Chinese digital periodical and conference database, China National Knowledge Infrastructure database, and the VIP Chinese Science and Technique Journals Database (VIP) will be searched by us for relevant literature. We will search for data in the above 9 Chinese and English databases, and the search time will from their inception to February, 2021. The key words include “tongxieyaofang,” “sinisan,” “irritable bowel syndrome,” “Irritable Syndrome,” “IBS,” and “random allocation.” We will also search ongoing or unpublished trials from the National Institutes of Health clinical registry Clinical Trials, International Clinical Trials Registry Platform and the Chinese clinical trial registration platform. PubMed's search strategy is shown in Table 1.

**Table 1:** Search strategy used in PubMed database.

Order	Search items
#1	(“Irritable Bowel Syndromes”[Mesh]) OR ((((((((Irritable Bowel Syndromes[Title/Abstract]) OR (Syndrome, Irritable Bowel[Title/Abstract])) OR (Syndromes, Irritable Bowel[Title/Abstract])) OR (Colon, Irritable[Title/Abstract])) OR (Irritable Colon[Title/Abstract])) OR (Colitis, Mucous[Title/Abstract])) OR (Colitides, Mucous[Title/Abstract])) OR (Mucous Colitides[Title/Abstract])) OR (Mucous Colitis[Title/Abstract])
#2	(((((tongxieyaofang[Title/Abstract]) OR (Tong-Xie-Yao-Fang[Title/Abstract])) OR (TXYF[Title/Abstract])) OR (Chinese herbal medicine[Title/Abstract])) OR (traditional Chinese medicine[Title/Abstract])) OR (TCM[Title/Abstract])
#3	(((((sinisan[Title/Abstract]) OR (Si-Ni-San[Title/Abstract])) OR (SNS[Title/Abstract])) OR (Chinese herbal medicine[Title/Abstract])) OR (traditional Chinese medicine[Title/Abstract])) OR (TCM[Title/Abstract])
#4	randomized controlled trial[Publication Type] OR randomized[Title/Abstract] OR placebo[Title/Abstract]
#5	#1 AND #2 AND #3 AND #4

Mesh = medical subject headings, TCM = traditional Chinese medicine.

### 3.3 Inclusion criteria for research selection

#### 3.3.1 Types of studies

All RCTs of TXYF combined with SNS in the treatment of IBS, whether blinded or unblinded.

#### 3.3.2 Type of participants

All patients diagnosed with IBS based on specific diagnostic criteria (Rome I criteria, Rome II criteria, Rome III criteria or Rome IV criteria).

#### 3.3.3 Type of interventions

The treatment of the experimental group is TXYF combined with SNS. The mode of administration is oral, and the dosage form is decoction or granule.

#### 3.3.4 Type of comparators

The control group was treated with conventional drugs or placebo.

#### 3.3.5 Types of outcome measures

The primary outcome measure is total effective rate. The secondary outcome measures include symptom score, functional digestive disorder quality of life scale, etc.

### 3.4 Exclusion criteria

(1) Non-RCT literature.
(2) Animal experiments, case reports and reviews, etc.
(3) Repeatedly detected or published literature.
(4) Secondary IBS caused by other underlying diseases
(5) Unable to obtain complete data or full text literature.

### 3.5 Selection of studies and data extraction

**Figure 1:**
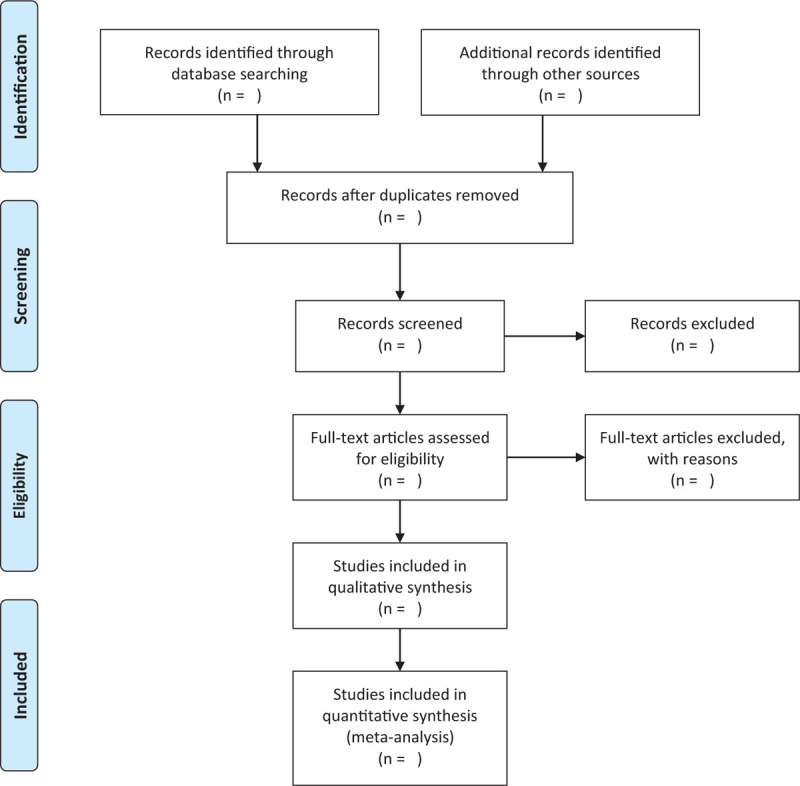
Flow chart of study selection.

Two researchers independently screened the literature. First, import the retrieved documents into EndNote X9 software to eliminate duplicate documents. Then the 2 researchers read the title and abstract, excluding obviously irrelevant documents. For uncertain documents, further read the full text to determine whether to include in the study. If there are inconsistent views, a third researcher will participate in the discussion and make the final decision. The selection process will be shown through the Preferred Reporting Items for Systematic Review and Meta-Analysis Protocols flow chart (Fig. 1).

In addition, two independent researchers extracted data from qualified literature according to a pre-designed data extraction table. The extracted content included the author's name, publication year, patient age, gender, diagnostic criteria, intervention measures, outcome indicators, and effect size.

### 3.6 Risk of bias assessment

The Cochrane collaborative tools will be used to evaluate the quality of the literature.^[19]^ The risk assessment of bias includes seven aspects: random sequence generation; hidden grouping; blinding to the research objects and treatment plan implementers; blinding to the outcome measurers; incomplete data; selective reporting of research results; other biases. For each research result, make low-risk, high-risk and unclear judgments on the above 7 items. The quality evaluation is carried out independently and in parallel by 2 evaluators. In case of inconsistent evaluation results, they must be resolved after discussion.

### 3.7 Quantitative data synthesis and statistical methods

#### 3.7.1 Quantitative data synthesis

The Review Manager (RevMan) V.5.3 software will be used for statistical analysis. When calculating the effect size, the relative risk and 95% confidence intervals are used for the dichotomous outcomes, and the weighted mean difference or standard mean difference will be used for the continuous outcomes.

#### 3.7.2 Assessment of heterogeneity

Chi-square test was used to test the heterogeneity among the studies, and the test level was α = 0.1. *I*^*2*^ test were used to estimates the degree of heterogeneity. When *I*^*2*^ < 40%, it indicates that no significant statistical heterogeneity. When 40% < *I*^*2*^ < 75%, it indicates that statistical heterogeneity, and the source of heterogeneity needs to be further analyzed. When *I*^*2*^ > 75%, it indicates that there is statistics heterogeneity between the studies.

#### 3.7.3 Assessment of reporting biases

Fewer than 10 studies do not require analysis of reporting bias. If more than 10 studies are included, we will use the symmetry of the funnel chart to detect potential reporting bias.^[20]^

#### 3.7.4 Subgroup analysis and Sensitivity analysis

If there is significant heterogeneity between the research results, we will conduct a subgroup analysis to investigate the differences in age, gender, etc. And we will also use sensitivity analysis to evaluate the robustness of analysis results.

#### 3.7.5 Grading the quality of evidence

Two researchers used the Recommendation, Evaluation, Development, and Evaluation system to independently evaluate the quality of evidence for each result. The Grades of Recommendation, Assessment, Development, and Evaluation system divides the level of evidence into 4 levels: high, medium, low, and very low.^[21]^

### 3.8 Ethics and dissemination

The study does not involve the personal information of participants, so no ethical approval is required. The research will be published in peer-reviewed journals or related journal meetings. The research results will provide new potential treatment strategies for patients with IBS.

## 4 Discussion

IBS has a high incidence worldwide, and there is still a lack of effective treatment measures. TXYF and SNS are used in the treatment of various gastrointestinal diseases in China. Studies have shown that they can regulate the intestinal flora, improve intestinal inflammation, etc.^[22,23]^ Although RCTs have shown that TXYF combined with SNS is effective in the treatment of IBS, there is no systematic evaluation and it has not been recognized internationally. Therefore, this study conducted a comprehensive systematic review and meta-analysis of the effectiveness of TXYF combined with SNS in the treatment of IBS, aiming to provide the latest evidence for the treatment of IBS and guide clinical decision-making.

## Author contributions

**Conceptualization:** Jiawang Jiang, Jing Ye.

**Data curation:** Ziyi Hu, Huaiyu Li, Haiyi Tang.

**Formal analysis:** Zhiying Yu, Yun Chen.

**Methodology:** Haiyi Tang, Ziyi Hu, Yun Chen.

**Software:** Zhiying Yu, Jiawang Jiang.

**Supervision:** Jing Ye, Huaiyu Li.

**Writing – original draft:** Jiawang Jiang, Huaiyu Li, Haiyi Tang.

**Writing – review & editing:** Jiawang Jiang, Jing Ye, Haiyi Tang, Zhiying Yu.
